# Zika Virus Inoculation During Pregnancy Impaired Maternal Care and Altered Prolactin and Corticosterone Levels in Rats

**DOI:** 10.1002/dneu.70053

**Published:** 2026-07-10

**Authors:** Meirylanne Gomes‐da‐Costa, Adriana Souza dos Santos, Anna Luísa Lothhammer Bohn, Bruna Carolina de Castro Saturnino, Gabrielle Batista de Aguiar, Gabriela Barth Jacobs, Chris Krebs Danilevicz, Ana Paula Muterle Varela, Thais Fumaco Teixeira, Paulo Michel Roehe, Lenir Orlandi Pereira

**Affiliations:** ^1^ Programa de Pós‐Graduação em Neurociências, Instituto de Ciências Básicas da Saúde Universidade Federal do Rio Grande do Sul Porto Alegre Rio Grande do Sul Brazil; ^2^ Departamento de Ciências Morfológicas, Instituto de Ciências Básicas da Saúde Universidade Federal do Rio Grande do Sul Porto Alegre Rio Grande do Sul Brazil; ^3^ Departamento de Farmacologia, Instituto de Ciências Básicas da Saúde Universidade Federal do Rio Grande do Sul Porto Alegre Rio Grande do Sul Brazil; ^4^ Departamento de Microbiologia, Imunologia e Parasitologia, Instituto de Ciências Básicas da Saúde Universidade Federal do Rio Grande do Sul Porto Alegre Rio Grande do Sul Brazil

**Keywords:** cortisol, dams, gestational ZIKV inoculation, HPA axis

## Abstract

Zika virus (ZIKV) is an *Orthoflavivirus* known for its teratogenicity and for causing congenital Zika syndrome (CZS) in the offspring, but recent studies suggest a negative impact on maternal brain health and behavior following gestational ZIKV inoculation. This behavior, crucial for the offspring's neurodevelopment, is regulated by the hypothalamic‐pituitary‐adrenal (HPA) axis and prolactin. This study investigated the effect of gestational ZIKV inoculation on maternal care in *Wistar* rats and its relationship with prolactin and corticosterone levels. Fifty‐six females were inoculated intraperitoneally on gestational day 9 (G9) with ZIKV (28 ZK group) or vehicle (28 CT group). Blood and hypothalamus samples from a set of these animals (*n* = 16/group) were collected 24 h postpartum to quantify corticosterone and prolactin levels by ELISA. Maternal behavior was evaluated on postnatal days 4 and 8 (*n* = 12/group). ZK rats demonstrated impaired maternal care compared to CT rats. Furthermore, 24 h postpartum, ZK rats had significantly lower levels of serum and hypothalamic corticosterone, and serum prolactin. The findings suggest that gestational ZIKV inoculation compromises maternal behavior in *Wistar* rats, concomitant with an alteration in the hormonal profile in the immediate postpartum period. The study is pioneering in demonstrating that ZIKV affects crucial components for the regulation of maternal behavior, reinforcing the need for further research to elucidate the mechanism in the maternal brain and develop targeted therapies.

## 1 Introduction

The Zika virus (ZIKV) is an *Orthoflavivirus* that can be transmitted by the *Aedes aegypti* mosquito bite, through vertical transmission, sexual contact, or blood transfusion, with the latter routes being less common (Agbajelola et al. 2025; A. Li et al. 2025). In 2015, there was an increase in congenital microcephaly cases due to ZIKV exposure in pregnant women (Martelli et al. 2024; Rabaan et al. 2025). Beyond microcephaly, gestational ZIKV infection can lead to several abnormalities, known as congenital zika syndrome (CZS), which comprise other outcomes such as cognitive impairments (Brasil et al. 2025; Marques et al. 2025). This viral epidemic, which started in the northeast of Brazil, resulted in a huge impact on public health and continues to require further investigation (Martins et al. 2025; Yuji Sasazaki et al. 2026).

Despite most of the literature focusing on ZIKV teratogenicity, little attention has been given directed toward the clinical repercussions for mothers. Oliveira et al. (2022) observed mothers of children with CZS for 5 years and identified signs of anxiety and depression among these parents. In a case‐control study with 213 postpartum women presenting depressive symptoms, a strong association was observed with more severe depressive symptoms and a diagnosis of ZIKV infection during pregnancy (Moraes et al. 2019). Thus, it is reasonable to hypothesize that ZIKV may affect maternal mental health, with significant gaps remaining regarding these processes.

Repercussions of ZIKV in mothers, in addition to causing harm to the mothers themselves, can also negatively impact their children. It is well established maternal effects from intrauterine neurodevelopment through the first years of life, impacting brain anatomy and physiology and the vulnerability of children to neurological disorders (Miguel et al. 2019). Through maternal care behavior, mothers assume a crucial role in the survival and neurodevelopment of their offspring. It is important to highlight that studies using animal models have already observed that changes in maternal care can lead to behavioral deficits and developmental disorders in the offspring (Bagheri and Goudarzi 2023; Gundacker et al. 2023; Yong et al. 2023). Deficient maternal behavior in *Wistar* rats has been associated with cognitive alterations in adult offspring (Potter et al. 2023). Conversely, interventions aimed at improving maternal care in rats can exert neuroprotective and anxiolytic effects on the offspring (Bagheri and Goudarzi 2023; Lorenzon et al. 2023).

Maternal behavior, and therefore maternal care, is influenced by a broad spectrum of physiological and pathological aspects, particularly throughout pregnancy. The regulation of maternal behavior occurs through hormones, such as prolactin and oxytocin, as well as through the control of the hypothalamic‐pituitary‐adrenal (HPA) axis, whose system participates in the synthesis of cortisol, which in humans is the main hormone involved in stress responses (Almanza‐Sepulveda et al. 2020; Daneshnia et al. 2024; Leff‐Gelman et al. 2025). Viral infections can dysregulate the HPA axis, resulting in the chronicity of high cortisol levels, present in some mood disorders, as well as an insufficient stress response (Alotiby 2024; George et al. 2025). Pathogens from viruses can induce neuroinflammation, culminating in neuropsychiatric and behavioral disorders, including maternal behavior (Depino 2006; Chou et al. 2024). Studies with animal models found that exposure to inflammation in the prenatal phase causes damage to the maternal behavior of rodents, which influences the offspring's brain development (Graciarena et al. 2010; Berger et al. 2018). Viral infections during pregnancy, such as influenza and SARS‐CoV‐2, suggest the existence of a relationship between maternal immune activation (MIA) and neurodevelopmental outcomes in the offspring (Couch et al. 2021; Han et al. 2021). Furthermore, inflammation and viral stress can, by themselves, induce or exacerbate mood disorders and cognitive dysfunctions in pregnant women (Park et al. 2024; Sahebi et al. 2024). Zambon et al. (2024), in their study on gestational infection with polyinosinic‐polycytidylic acid in mice, showed that MIA impaired hypothalamic circuitry and altered maternal care. Ronovsky et al. (2017) found similar findings, suggesting transgenerational effects on maternal care.

Although several preclinical studies published since 2016 have reported the teratogenic effects and mechanisms of prenatal ZIKV exposure, less attention has been given to the impacts of this infection on maternal care. While clinical reports describe these repercussions in mothers during and after pregnancy, only one published study using an animal model of gestational ZIKV infection (on gestational day 18) has addressed this topic (Dos Santos Oliveira et al. 2016; Kuper et al. 2019; Santos et al. 2021). However, this study evaluated maternal care only on the first postnatal day, and found no effect (Sherer et al. 2021).

The present study aimed to evaluate the maternal care of *Wistar* rats, at two time points, on postnatal days 4 and 8, that experienced ZIKV infection during pregnancy and to investigate whether prolactin and corticosterone levels in the hypothalamus and serum are related to maternal care behavior possibly affected by viral infection.

## 2 Materials and Methods

### 2.1 Animals

This study follows the norms of the Arouca Law (Law no. 11.794/2008) and the Guide for the Care and Use of Laboratory Animals used by the National Institute of Health (USA) and was approved by the ethics committee of the Federal University of Rio Grande do Sul, Brazil (No. 46378). Three‐month‐old pregnant *Wistar* rats (*N* = 56) were obtained from the Center for Reproduction and Experimentation of Laboratory Animals—CREAL, Federal University of Rio Grande do Sul, Brazil. The responsible veterinarian and her team confirm the presence of spermatozoa after mating before providing the animals for the study. All the dams were acclimated in standard bioterium conditions: 12/12‐h light/dark cycle, controlled temperature (22 ± 2°C), and free access to food and water.

**FIGURE 1:**
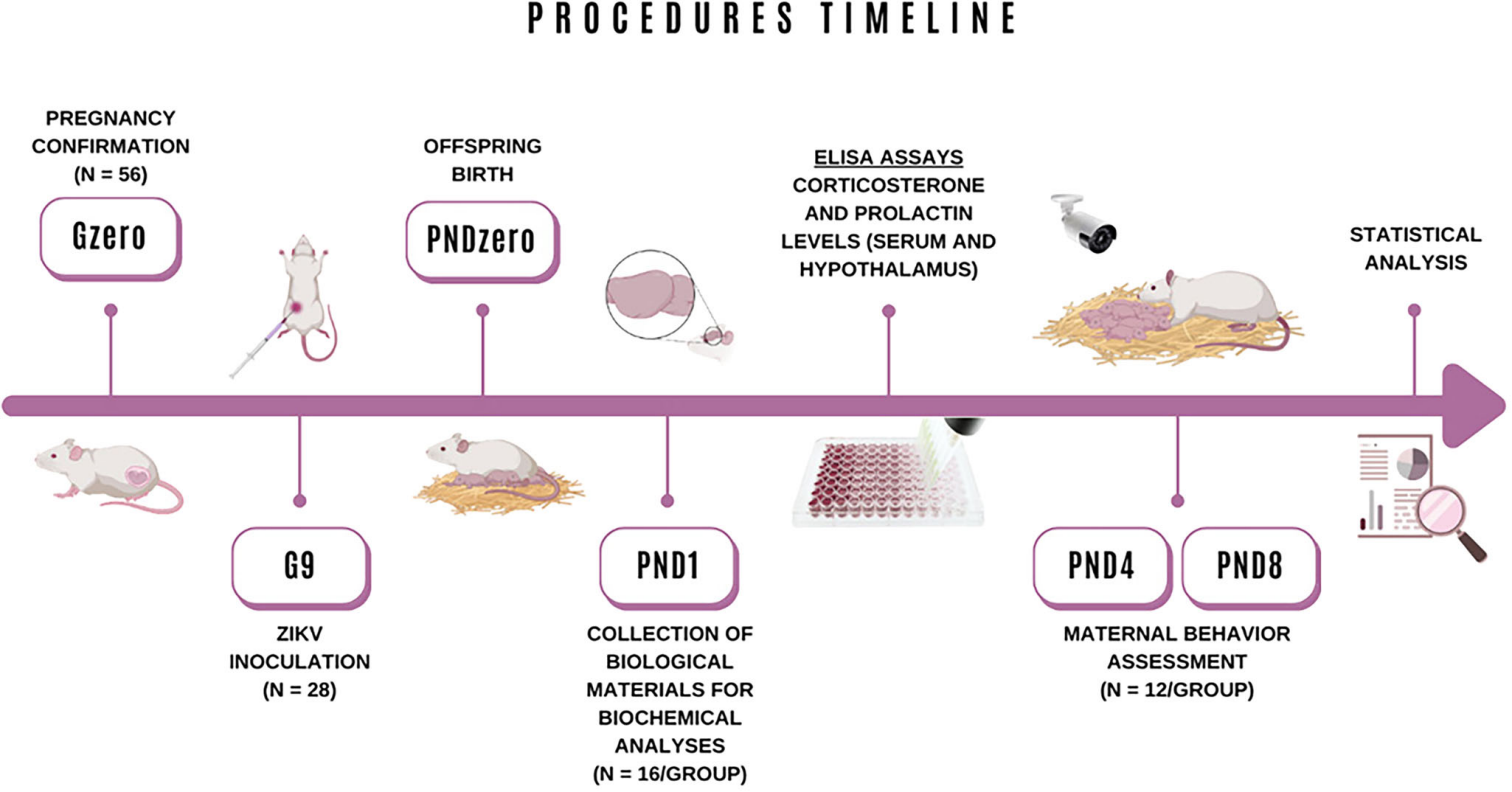
Procedures timeline—After pregnancy confirmation (G0), the dams were randomly assigned to either control group (CT) or zika group (ZK). On the gestational day 9 (G9), the control group received an intraperitoneal injection of sterile medium, while the ZK group was inoculated with ZIKV. Postnatal day 0 (PND0) was the day of offspring birth. On PND1, a set of dams had their biological materials collected. Subsequently, the ELISA was performed. The remained dams proceeded to the maternal behavior assessment. G, gestational day, PND, postnatal day, ZIKV, Zika virus. Image: own authorship with components from BioRender.com.

On gestational day 9 (G9), females were randomly assigned to two groups: females receiving an intraperitoneal injection of 500 µL of 1 × 10^6^ plaque‐forming unit (PFU mL^−1^) of ZIKV isolated in Brazil (ZIKV‐BR; *n* = 28) and the other group inoculated via intraperitoneal with 500 µL of innocuous medium (*n* = 28) (Figure 1). The G9 was chosen for viral inoculation since it corresponds to the first trimester in human gestation, which is marked by the start of neurodevelopment (de Almeida et al. 2023). All handling of the ZIKV and control groups was performed in a biological safety cabinet (Tecniplast BS 60 class II, Buguggiate, Italy). All animals were acclimated in “Individually Ventilated Cages” (IVC; 904 cm^2^ area, 18 cm height, Tecniplast, Buguggiate, Italy). Litters’ birth was considered PNDzero and were standardized on PND1 with four males and four females neonate rats for each dam. The excess neonates were euthanized.

### 2.2 Euthanasia and Collection of Biological Samples

Dams that were not submitted to maternal behavior evaluation were decapitated 24 h postpartum (*n* = 16/group), and their hypothalamus and blood samples were collected. The hypothalamus of each dam was dissected and placed in Eppendorf tubes, then placed in liquid nitrogen. The blood was immediately placed in Eppendorf tubes containing heparin and subsequently centrifuged in a refrigerated centrifuge at 4000 rpm at 4°C for 15 min. After, the serum (supernatant) was collected and properly cryopreserved. Body (*N* = 23) and adrenals weight (*N* = 24) were also measured using a high precision balance (Marte AD4200), and the data were recorded. Immediately after collection, all samples were stored at −80°C for biochemical analyses.

### 2.3 Corticosterone and Prolactin Levels

Hormone levels (corticosterone: *n* = 8/group; prolactin: *n* = 7/group) in the dam's serum and hypothalamus were measured using ELISA kits (FineTest, Wuhan, China), all procedures performed according to the manufacturer's instructions, utilizing recommended samples dilutions and standard curve concentrations: corticosterone (catalog No. ER0859) and prolactin (catalog No. ER0076). For this assay, hypothalamic tissue was homogenized in PBS 1% protease inhibitor cocktail (PIC) centrifuged at 4000 rpm at 4°C for 15 min. The resulting supernatant and serum samples were added to a diluent reagent in a 96‐well plate and incubated overnight at 4°C with 100 µL of capture antibody (diluted). After washing, 100 µL of either samples or standards were added and incubated for 60 min at 37°C. Then, a working solution was added, incubated for 30 min at 37°C and the wells were replaced with 90 µL of substrate solution and incubated for 20 min at 37°C. Finally, 50 µL of stop solution was added, and absorbance was immediately measured at 450 nm using a microplate reader. Results were expressed as ng/mL (protocol adapted from Dos Santos et al. 2025).

### 2.4 Maternal Behavior Assessment

In this study, maternal behavior was evaluated on PND4 and PND8 (*n* = 12 dams/group) through 15‐min video recordings for each animal, during which continuous observation was performed. These recordings were registered during the light cycle on cage‐cleaning days, a period when maternal behaviors are more prominently expressed. The observed variables were the latency to start nesting, the latency to retrieve the first pup, and the total time the dams spend in the nest with their pups (Dos Santos et al. 2023). When the rat cages were changed for cleaning, the pups were placed scattered among the wood shavings. Subsequently, the dam was returned to the cage, and behavioral recordings were initiated immediately. The separation time for of the pups from their dams was less than a minute. During the observation of maternal care, the time taken by the dam to pick up the first pup and return it to the nest was recorded. Nesting behavior was considered to have started when the dam chose a spot in the cage and started digging, thus establishing an area where she would later place all the pups. All analyses were performed by a blinded observer.

### 2.5 Statistical Analysis

To find out whether the data were normally distributed, the Shapiro–Wilk normality test was applied. *T*‐test was used for maternal care data, body and adrenals weight, hypothalamic corticosterone, serum prolactin, and hypothalamic prolactin. Mann–Whitney test was applied for serum corticosterone. A significance level of *p* < 0.05 for all statistical analyses. For the body weight analysis, the sample sizes were *n* = 11 for the CT group and *n* = 12 for the ZK group.

The programs used for statistical analysis were The R Project for Statistical Computing (version 4.5.1) and GraphPad Prism (version 9.0).

## 3 Results

### 3.1 Body Weight and Adrenals Weight

No differences were found between the CT and ZK groups in body weight [*t*(21) = 0.366, *p* = 0.718] and adrenals weight [*t*(22) = 1.366, *p* = 0.186] (Table 1).

**TABLE 1:** Analysis of the body weight (*N* = 23: 11 CT group, 12 ZK group) and adrenals weight (*N* = 24: 12/group).

	CT group	ZK group
Body weight (g)	262.91 ± 4.831	260.80 ± 3.497
Adrenals weight (g)	0.0821 ± 0.0020	0.0777 ± 0.0025

*Note*: Data are expressed as mean ± SEM. ZK: dams inoculated with the Zika virus during pregnancy. CT: dams of the control group.

*p ≤ 0.05, Student *T*‐test.

### 3.2 Corticosterone and Prolactin Levels

#### 3.2.1 Serum and Hypothalamic Corticosterone

**FIGURE 2:**
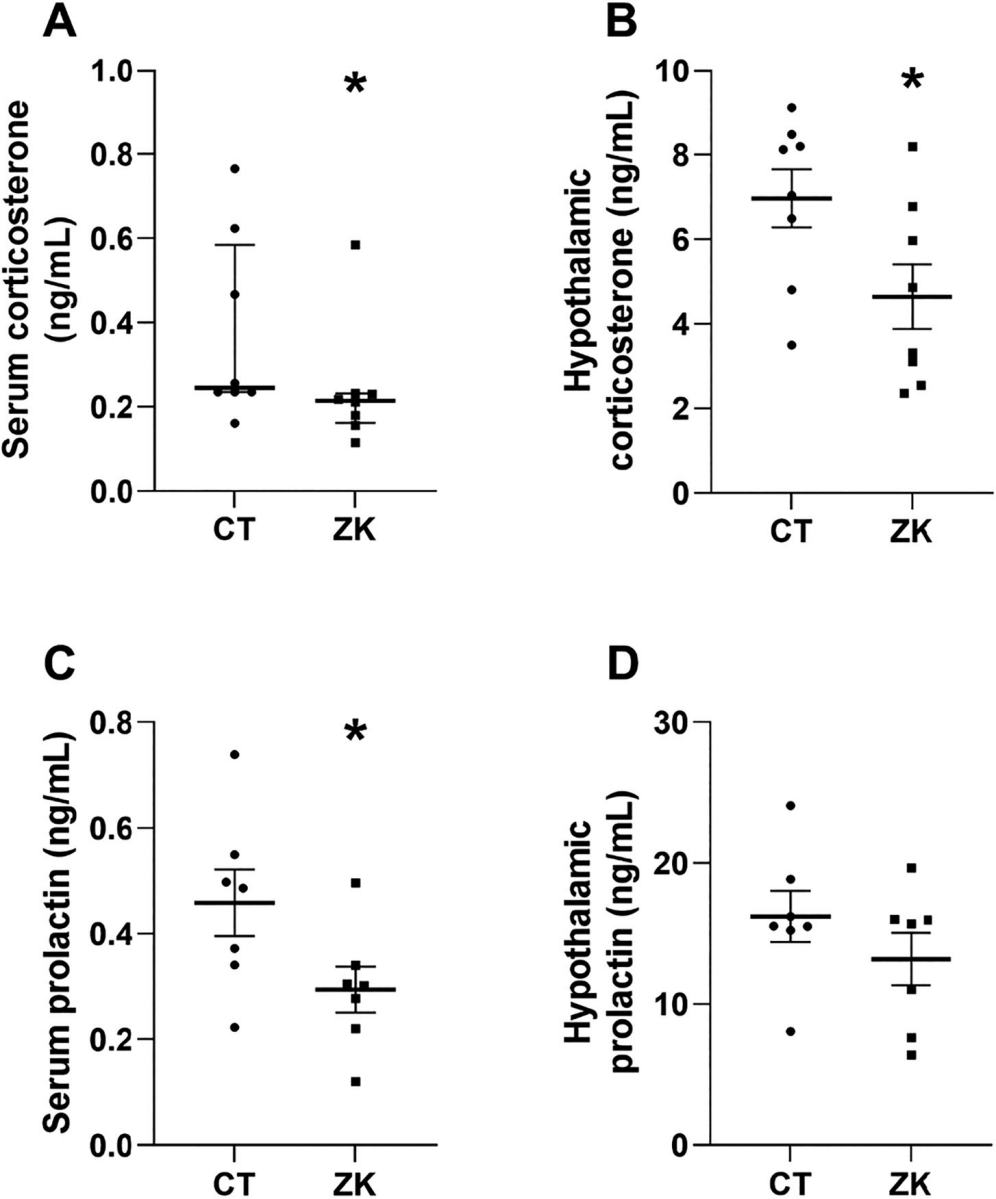
(A) Serum corticosterone levels in female *Wistar* rats. Values represent median and interquartile ranges. *p < 0.05, Mann–Whitney test (*n* = 8). (B) Corticosterone levels in the hypothalamus of female *Wistar* rats. Values represent the mean and standard error of the mean (SEM). *p < 0.05, *T*‐test (*n* = 8). (C) Prolactin levels in the serum of female *Wistar* rats. Values represent mean and SEM. *p < 0.05, *T*‐test (*n* = 7). (D) Prolactin levels in the hypothalamus of female *Wistar* rats. Values represent mean and SEM. *p < 0.05, *T*‐test (*n* = 7).

To analyze corticosterone levels in blood serum, the Mann–Whitney test was applied. The results showed lower serum levels in the ZK group when compared to the CT group (*U* = 11; *p* = 0.02) (Figure 2A).

The *t*‐test demonstrated that ZK group exhibited lower corticosterone levels in the hypothalamus compared to the CT group [*t*(14) = 2.261, *p* = 0.04] (Figure 2B).

#### 3.2.2 Serum and Hypothalamic Prolactin

*T*‐test revealed lower serum prolactin levels in the ZK group than the CT group [*t*(12) = 2.146, *p* = 0.05] (Figure 2C). No differences were found in prolactin levels in the hypothalamus between the CT and ZK groups [t(12) = 1.163, *p* = 0.26] (Figure 2D).

### 3.3 Maternal Behavior Assessment

**FIGURE 3:**
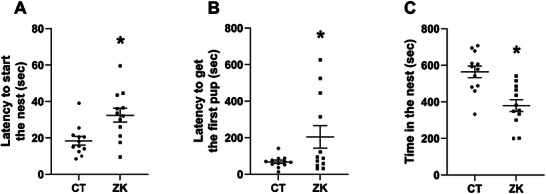
Variables analyzed in maternal care (*n* = 12). (A) Latency to start the nest. (B) Latency to get the first pup. (C) Time in the nest. Data are expressed as mean ± SEM. Data from PND 4 and PND 8 were pooled for each group. ZK: dams inoculated with the Zika virus during pregnancy. CT: dams of the control group. Student *T*‐test, *p ≤ 0.05.

Student *T*‐test identified that ZK dams took longer to start the nest [*t*(22) = 3.117, *p* = 0.005], more time catching their first pup [*t*(22) = 2.188, *p* = 0.039] and less time in the nest [*t*(22) = 4.046, *p* < 0.001], when compared to the CT animals. The results clearly showed an impaired quality of maternal care in the ZK group (Figure 3).

## 4 Discussion

Since 2015, following the ZIKV epidemic, particularly in South America, CZS has been studied. Most research has focused on offspring development, which can be profoundly impacted by gestational infection. However, the effects of the virus on the parents, particularly on maternal behavior, have been rarely investigated. The present study aimed to evaluate maternal care of *Wistar* rats that experienced ZIKV inoculation during pregnancy and to investigate whether prolactin and corticosterone levels, in the hypothalamus and serum are related to maternal care behavior potentially affected by viral infection. Our data clearly revealed an impairment in maternal care because of gestational viral inoculation. Decreased corticosterone and prolactin levels were found in these dams.

It is known that gestational complications, such as infections during pregnancy, could lead to changes in maternal care (Ronovsky et al. 2017). Our findings demonstrated that ZIKV inoculation on the gestational day 9 (G9) leads to impairment in maternal care during the postnatal period. Such changes may be associated with ZIKV infection, whose pathogen causes an inflammatory response generating stress and consequent homeostatic imbalance (Chudnovets et al. 2020). Exposure to stressors in the prenatal phase triggers neuroendocrine dysfunctions, possibly impairing maternal behavioral experiences (Bridges 2015). It is interesting to highlight that maternal care is important for the neurodevelopment and survival of offspring and its changes influence their behavior (Yong et al. 2023). It is well established that a range of viral infections, such as ZIKV infection, during pregnancy negatively impact the offspring, which may present structural and functional abnormalities of the nervous system (Vadini et al. 2016; Auriti et al. 2021; De Rose et al. 2023). However, it is rarely explored the impact of viral infection on maternal behavior. Our rat model was the first to identify the effects of maternal ZIKV inoculation on maternal care, as well as the long‐term effects on the offspring of these rats, thus contributing to the standardization of a rat model of congenital syndrome associated with ZIKV infection (da Costa et al. 2025). These findings contribute not only to understanding the repercussions of ZIKV infection, but also to making it feasible to develop therapeutic plans for pregnant women affected by this clinical condition. Although we demonstrated impairment in maternal care, the mechanism by which these changes occur remains unknown. Based on these findings, we hypothesize that gestational ZIKV infection acts as a potent biological and immunological stressor, leading to persistent neuroinflammation in the maternal brain, which possibly causes dysfunction of the HPA axis and the neuro‐hormonal prolactin circuit. This imbalance may lead to a failure in the regulation of maternal behavior. Therefore, more studies with maternal ZIKV infection and evaluation of maternal care are needed aiming to elucidate the mechanism of ZIKV in the dams' brain.

Some neuroendocrine components are responsible for maintaining the homeostasis of maternal behaviors, with prolactin and glucocorticoids—such as cortisol in humans and corticosterone in rodents—playing a central role in their regulation (Brummelte and Galea 2016; Bridges 2020). Physiologically, an increase in prolactin levels is expected in the immediate postpartum period for the mother to initiate and maintain maternal behaviors (Brown et al. 2017; Chasseloup et al. 2024). Our data revealed low prolactin levels in the ZK group 24 h postpartum; such effect was identified in serum but not in the hypothalamus. Prolactin also is an important hormone of maternal behavior in rodents, its acts on prolactin receptors in the hypothalamus that is essential for the beginning of interactions with the pups and the maintaining maternal care (Georgescu et al. 2021). However, this reduction in the serum prolactin levels, as our results suggest, may have been caused by interference with its secretion by the anterior pituitary gland or in its transport across the blood–brain barrier, compromising the maintenance of appropriate maternal behavior (Larsen and Gratta 2012). These findings suggest the virus‐induced hormonal dysfunction, closely associated with maternal care. The relationship between viral infections and hormonal responses during pregnancy has been studied for Hepatitis viruses (M. Li et al. 2024) and SARS‐CoV (Ntounis et al. 2022). Additionally, the interaction between the immune system and maternal hormone levels—particularly prolactin—is already well established. Therefore, it is reasonable to consider that viral inoculation during pregnancy triggers an immune response which, due to the unique hormonal environment of gestation, may be inadequate or dysregulated, ultimately leading to alterations in maternal hormones such as prolactin. Taken together, these data support the notion that prolactin dysregulation may partially underlie the impaired maternal care observed.

Viral infection in pregnancy, such as that caused by the ZIKV, besides inducing an inflammatory response, can trigger stress condition that typically elevates the activity of the HPA axis, resulting in an increase in glucocorticoids (Knezevic et al. 2023). We hypothesize that ZIKV inoculation could increase corticorterone levels, in progenitors. However, surprisingly, our data revealed low levels of corticosterone in the ZK dams. Hypocortisolism, in humans, is a condition found in some chronic infections and adrenal dysfunction (Kunugi et al. 2015; Leow et al. 2005; Wolff et al. 2023). Alternatively, these low glucocorticoid levels may indicate relative adrenal insufficiency or HPA axis dysregulation induced by the virus or by a prolonged inflammatory response, thereby impairing neurochemical homeostasis (Varghese et al. 2016). In this context, a recent study revealed that hepatitis virus was responsible for a decreased cortisol in serum (Zhang et al. 2023). At this stage, it is not possible to conclude that a stress condition is present as a consequence of gestational viral inoculation. On the other hand, reduced activation of the HPA axis could contribute to diminished maternal responses.

The study was pioneering, adopting a model of CZS in *Wistar* rats during early pregnancy, demonstrating an impairment in maternal behavior associated to decreased prolactin and corticosterone levels in the dams 24 h postpartum. Although the exact mechanism of this hormonal dysfunction and behavioral alteration is unknown, these findings suggest that ZIKV does not only affect the offspring, according to our previously published studies (da Costa et al. 2025; de Almeida et al. 2023), but also maternal brain health by disrupting neuroendocrine components crucial for the regulation of maternal behavior. These findings contribute to the understanding of the repercussions of ZIKV infection and reinforce the need for further studies to elucidate the ZIKV signaling pathway in the maternal brain, aiming at the development of targeted therapeutic plans also for affected pregnant women. Supplememtary Material

## Author Contributions

Meirylanne Gomes‐da‐Costa and Adriana Souza dos Santos were responsible for writing, resource management, methodology, investigation, formal analysis, data curation, and conceptualization. Bruna Carolina de Castro Saturnino and Gabrielle Batista de Aguiar focused on the experimental stage, investigation, and statistics. Chris Krebs Danilevicz contributed to the development of techniques and methodological review. Anna Luísa Lothhammer Bohn and Gabriela Barth Jacobs participated in the collection of biological materials and bench experiments. Ana Paula Muterle Varela, Thais Fumaco Teixeira, and Paulo Michel Roehe handled ZIKV manipulation and dosage. Lenir Orlandi Pereira participated in writing – review and editing, supervision of all stages of the scientific research, funding acquisition, formal analysis, data curation, and conceptualization. All authors read and approved this manuscript.

## Ethics Statement

All procedures follow the norms of the Arouca Law (Law no. 11.794/2008) and the Guide for the Care and Use of Laboratory Animals used by the National Institute of Health (USA) and was approved by the ethics committee of the Federal University of Rio Grande do Sul, Brazil (No. 46378).

## Conflicts of Interest

The authors declare no conflicts of interest.

## Supporting information




**Supplementary Materials**: dneu70053‐sup‐0001‐SuppMat.docx

## Data Availability

The data that support the findings of this study are available from the corresponding author upon reasonable request.
