# Supplementary material for: Zika Virus Inoculation During Pregnancy Impaired Maternal Care and Altered Prolactin and Corticosterone Levels in Rats
Source: Dev Neurobiol. 2026 Jul 10;86(3):e70053. doi: 10.1002/dneu.70053 (PMC13352443; doi:10.1002/dneu.70053)
Supplement: Supplementary file 1 — Supplementary Materials: dneu70053‐sup‐0001‐SuppMat.docx [file DNEU-86-0-s001.docx]

**SUPPLEMENTARY INFORMATION FOR REVIEW**

**Transparent Science Questionnaire for Authors**

Section: **3. Experimental Subjects**

**a)** Explain how the number of animals were arrived at and provide details of any sample size calculation, including power analysis.

**We considered the studies by Pereira et al. (2007), and Schuch et al. (2016) to perform the sample size calculation for the behavioral analysis. For the ELISA analysis, we based our estimate on the study by Dos Santos et al. (2023).**

**The calculation was performed using the tool available at: Cálculo Amostral - Estatística ([Cálculo Amostral - Estatística](http://calculoamostral.bauru.usp.br/calculoamostral/index.php) ). Considering 80% of power analysis.**

DOS SANTOS, Adriana Souza et al. Resistance exercise was safe for the pregnancy and offspring’s development and partially protected rats against early life stress-induced effects. Behavioural Brain Research, v. 445, p. 114362, 2023.

PEREIRA, L. O. et al. Effects of daily environmental enrichment on memory deficits and brain injury following neonatal hypoxia-ischemia in the rat. Neurobiology of Learning and Memory, v. 87, n. 1, p. 101–108, jan. 2007.

SCHUCH, C. P. et al. Early environmental enrichment affects neurobehavioral development and prevents brain damage in rats submitted to neonatal hypoxia-ischemia. Neuroscience Letters, v. 617, p. 101–107, 23 mar. 2016.

Section: **4. Data Handling**

**a)** Define the criteria for data/subject inclusion and exclusion. If any outcome or condition measure used was not reported in the results section, authors must address this omission.

**Inclusion: Pregnant 3-month-old *Wistar* rats.**

**Exclusion: Pups were standardized to four males and four females; excess neonates were euthanized. The animals were divided for different purposes: one set for sample collection 24 hours postpartum, and another for maternal behavior assessment.**

Section: **5. Statistical Analysis and Depiction of Continuous Data**

**c)** Fully report statistics (including exact value of N, degrees of freedom, test value and exact P-value when >0.001) and we encourage the use of effect sizes and confidence intervals.

**Complete statistics are provided, such as: Body Weight [t(21) = 0.366, p = 0.718]. Hypothalamic Corticosterone [t(14) = 2.261, p = 0.04]. Time in Nest [t(22) = 4.046, p < 0.001]. The N value is indicated in all Results sections and Figures (e.g., n=8, n=7, n=12).**
